# Meta-analytic Techniques to Assess the Association Between N-acetylcysteine and Acute Kidney Injury After Contrast Administration: A Systematic Review and Meta-analysis

**DOI:** 10.1001/jamanetworkopen.2022.20671

**Published:** 2022-07-05

**Authors:** Kate Magner, Julius Vladimir Ilin, Edward G. Clark, Jennifer W. Y. Kong, Alexandra Davis, Swapnil Hiremath

**Affiliations:** 1 Department of Medicine, University of Ottawa, Ottawa, Ontario, Canada; 2 Kidney Research Institute, Ottawa Hospital Research Institute, Ottawa, Ontario, Canada

## Abstract

**Importance:**

The most suitable analytic method to systematically analyze numerous trials with contradictory results is unclear. Multiple trials assessing the use of N-acetylcysteine (NAC) for prevention of contrast-induced acute kidney injury (CI-AKI) have had contradictory results with recent trials confirming a lack of benefit.

**Objective:**

To systematically review the literature on NAC for the prevention of CI-AKI, and to explore the heterogeneity, publication bias, and small-study effect to determine the most suitable analytic method in a setting where the literature is contradictory.

**Data Sources:**

Medline, Embase, and Cochrane Central Register of Controlled Trials databases were used to find randomized clinical trials (RCTs) comparing NAC with any other prophylactic agent or placebo in adults.

**Study Selection:**

The search included studies published in English from database inception to January 2020. Two independent reviewers screened the studies, extracted data, and performed the risk of bias assessment.

**Data Extraction and Synthesis:**

A meta-analysis was conducted about the effect of NAC on CI-AKI, the need for dialysis, and mortality. Fixed and random effects analyses were also performed. Funnel plots and the trim and fill method were used for assessment of publication bias. Metaregression was performed to explore the heterogeneity and subgroup analysis to examine the association between NAC and CI-AKI when studies were categorized according to sample size and number of events.

**Results:**

A total of 101 trials were included in this meta-analysis. The median sample size was 112 (range, 20 to 4993). Twenty-nine trials had a sample size of 200 or more, and only 3 trials had a sample size of 500 or more. Forty-five trials reported the need for kidney replacement therapy, and 41 trials reported mortality as an outcome. NAC seemed to show a benefit, with a pooled OR of 0.72 (95% CI, 0.63-0.82) using random effects model and a pooled OR of 0.82 (95% CI 0.76-0.90) using a fixed effects model. However, there was significant heterogeneity (*I^2^* = 37.6; *P* < .001) and significant publication bias, which was reduced only when restricting to large RCTs (N ≥ 500). The clinical outcomes (ie, the need for kidney replacement therapy and mortality) revealed little heterogeneity and no publication bias, and each provided a robust neutral summary result.

**Conclusions and Relevance:**

In this meta-analysis, NAC was associated with a benefit in the prevention of CI-AKI. However, because of substantial publication bias and other biases, standard meta-analytic techniques resulted in significant heterogeneity and a spurious, or factitious, association, even when using a random effects model. When the analysis was restricted to RCTs with a large sample size to account for publication bias or restricted to trials with clinical outcomes, this issue was reduced and resulted in more robust and neutral effect sizes.

## Key Points

**Question:**

What is the association between N-acetylcysteine (NAC) and acute kidney injury after contrast administration?

**Findings:**

In this meta-analysis of 101 randomized control trials, NAC was associated with the prevention of contrast-induced acute kidney injury when all studies were considered together using either a fixed-effects regression model or random effect analysis; however, there was substantial statistical heterogeneity and publication bias, which undermined the validity of these pooled estimates. Restricting the analysis to large trials (ie, trials with sample size greater than 500 or event rates greater than 100), or analyzing trials with clinical, not biochemical, outcomes reduced these issues and resulted in a null effect.

**Meaning:**

These findings suggest that meta-analyses should consider the influence of sample size and meaningful and clinically relevant outcomes in the setting of heterogeneous literature.

## Teaser

This systematic review and meta-analysis of N-acetylcysteine for the prevention of contrast-imaging acute kidney injury explores the heterogeneity, publication bias, and small study effect to determine the most suitable analytic method in a setting where the literature is contradictory.

## Introduction

Iodinated contrast is a potential cause of acute kidney injury (AKI) and subsequent morbidity and mortality in patients who receive contrast media for diagnostic and therapeutic procedures.^1^ Often mild and reversible, contrast-induced AKI (CI-AKI) is not significantly associated with a rise in markers of renal tubular injury and may reflect changes in kidney hemodynamics rather than true kidney damage.^1,2^ However, in a subset of patients, CI-AKI can cause clinically meaningful outcomes, including the progression of underlying chronic kidney disease, the need for kidney replacement therapy (KRT), prolonged hospitalization, and death. As a predictable iatrogenic event, CI-AKI should be avoidable and multiple strategies for the prevention of AKI have been evaluated in the literature. Among potential preventative agents, N-acetylcysteine (NAC) underwent the most trials in the last 2 decades (eTable 1 in the Supplement). These trials may be characterized as initially having shown promising results but led to large null trials and clear evidence for lack of efficacy.^3,4,5^

While equipoise no longer exists to use NAC in this setting, the field went through almost 2 decades of misleading findings and NAC use, exacerbated by mixed results from the multiple trials, many of which reported a benefit with NAC in prevention of CI-AKI.^4,6^ The mix of some trials showing a benefit with NAC and others reporting null findings has also resulted in the publication of many systematic reviews, most of which report a benefit of NAC, and we now know that this is an inaccurate finding.^7,8^

We undertook a systematic review of the literature on NAC for the prevention of CI-AKI to explore how heterogeneity, publication bias, and small-study effects shape meta-analytical outcomes. In so doing, we aimed to determine the most suitable analytic method in a setting where the literature is contradictory.

## Methods

The systematic review and protocol were registered with the PROSPERO registry CRD42020217903. This study followed the Preferred Reporting Items for Systematic Reviews and Meta-analyses (PRISMA) reporting guideline.^9,10,11^

### Search Strategy and Study Selection

We searched Medline, Embase, and Cochrane Central Register of Controlled Trials databases for randomized clinical trials (RCTs) published from the database inception until January 2020. Studies were included if they were conducted in adults, published in English, and compared NAC with any other prophylactic agent or placebo or control for the prevention of CI-AKI in the setting of contrast administration. The search was designed by an information specialist (A.D.), and the details of the search strategy can be seen in the eTable 1 in the Supplement. Two independent reviewers (K.M. and J.I.) screened titles and abstracts for eligibility. The reviewers then undertook a second screening of the full text articles to confirm their eligibility. Any disagreements were resolved by discussion and consensus.

### Data Extraction

A data extraction template was developed by an independent reviewer and modified by feedback from the other investigators. The 2 reviewers extracted prespecified data points from each full text article and compared them for consistency, resolving discrepancies with consensus. The following information was extracted from all the included studies: study characteristics, setting of contrast administration, CI-AKI definition, sample size, study population characteristics, NAC dose and route, control intervention, mean precontrast and postcontrast creatinine, and outcomes, including rate of CI-AKI, dialysis, and death.

### Risk of Bias Assessment

Risk of bias assessment was undertaken by the 2 reviewers using the Cochrane Collaboration tool for assessing the risk of bias in randomized control trials.^12^ Disagreements between reviewers were resolved by discussion and consensus.

### Statistical Analysis

The primary outcome was CI-AKI, using the definitions used in the individual studies. Secondary outcomes were the need for kidney replacement therapy (KRT) and all-cause mortality.

Using the extracted data, we calculated summary odds ratios (OR) and 95% CIs for the association of NAC with the prevention of CI-AKI, the need for KRT, and mortality. Association size was calculated and presented using both a fixed effects model and a random effects model, using the method described by DerSimonian and Laird.^13^ To evaluate heterogeneity across the included trials, we used the Cochran Q statistic test and the *I^2^* statistic, with statistical significance set at *P* < .10. A funnel plot, the Egger statistic, and the Duval-Tweedie trim and fill method were used to assess publication bias.^14,15^ Subgroup analyses were performed on the basis of study size (defined on basis of sample size threshold of 200 and 500 and total events reported of greater or less than 100), and route and dose of NAC, with the aim of resolving heterogeneity and publication bias. Univariate metaregression was conducted at study level continuous variables (ie, mean age, proportion of men and diabetes, year of publication, NAC dose, baseline and change in creatinine, route of contrast, total sample size, and event numbers). Statistical significance was set at *P* < .05, and tests were 2-sided. All analysis was conducted with Comprehensive Meta-analysis version 3.3.070 (Biostat Inc).

## Results

The electronic database search identified 654 citations. Of these, 223 full texts were assessed for eligibility, and 122 were excluded because they did not meet the eligibility criteria (Figure). In total, 101 (15%) trials were included in the meta-analysis. Summary study characteristics are shown in Table 1, and full trial details are summarized in the eAppendix in the Supplement.

**Figure.**
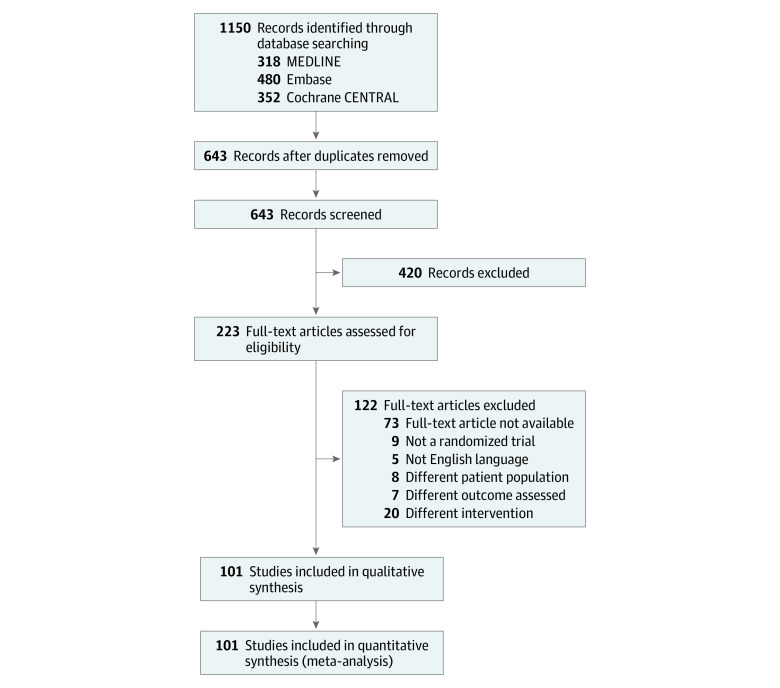
Study Flowchart

**Table 1.** Study Characteristics

Characteristics	No. (%)
Total studies, No.	101 (100)
Contrast setting	
Arterial (diagnostic and/or therapeutic)^a^	85 (84)
Venous contrast^b^	16 (16)
NAC route	
Oral	67 (66)
Intravenous^c^	26 (26)
Oral and IV	4 (4)
Total NAC dose	
<2400 mg	17 (17)
2400 mg	48 (48)
>2400-4800	20 (20)
>4800 mg	16 (16)
Sample size	
<200	72 (71)
≥200 to <500	26 (26)
≥500	3 (3)
CI-AKI definitions (increase in creatinine)	
0.5 mg/dL or 25% in 48 h	35 (35)
0.5 mg/dL 48 h	11 (11)
25% in 48 h	14 (14)
25% in 48-72 h	2 (2)
0.5 mg/dL or 25% in 48-96 h	2 (2)
25% in 72 h	9 (9)
0.5 mg/dL in 72 h	2 (2)
0.5 mg/dL or 25% in 72 h	7 (7)
0.5 mg/dL in 5 d	1 (1)
25% in 5 d	1 (1)
0.3 mg/dL or 20%	3 (3)
25% in 48-96 h	1 (1)
0.5 mg/dL or 25% in 48-72 h	7 (7)
0.5 mg/dL or 25% in 24-72 h	1 (1)
0.3 mg/dL in 48 h or 50% after 7 d	1 (1)
Increase in cystatin-C by 25% in 72 h	1 (1)
Not specified	3 (3)
Control intervention^d^	
Normal saline and placebo	38 (38)
Placebo	51 (50)
Sodium bicarbonate	6 (6)
Others^e^	10 (10)

Abbreviations: CI-AKI, contrast induced acute kidney injury; IV, intravenous; NAC, N-acetylcysteine.

SI conversion factor: To convert creatinine to μmol/L, multiply by 88.4.

^a^ Arterial includes coronary angiogram (N = 41); coronary angiogram without percutaneous coronary intervention (N = 34); coronary and peripheral angiogram (N = 6); peripheral angiogram (N = 4); endovascular aortic aneurysm repair (N = 1).

^b^ Venous includes computed tomography scans (N = 13); intravenous pyelogram (N = 1); computed tomography angiogram (N = 2).

^c^ Includes 1 study which also combined this with intrarenal NAC.

^d^ Four trials compared NAC with 2 different control arms and each of these was treated as separate data points, thus giving a total N = 106.

^e^ Others includes theophylline (N = 2), and 1 each of orange juice, fenoldopam, fenoldopam with normal saline, ascorbic acid, 5% dextrose, normal saline with cola, normal saline with statin, and normal saline with placebo with sodium bicarbonate.

Across all trials, sample sizes had a median (range) of 112 (20 to 4993), 29 trials (29%) had a sample size greater than or equal to 200, and 3 trials (3%)^3,5,16^ had a sample size of more than 500 participants. Thus, most of the trials (72 [71%]) included less than 200 participants. Similarly, only 6 trials^3,5,16,17,18,19^ (6%) had 100 or more events. The most common CI-AKI definition was a rise in creatinine of 0.5 mg/dL (to convert to micromoles per liter, multiply by 88.4) or 25% at 48 hours (35 [35%]). Variations of this (eg, only 25%, only 0.5 mg/dL, or a different timeframe ranging from 24 to 96 hours) accounted for the other definitions, with a smaller change (0.3 mg/dL) accounting for a small minority (Table 1).

The most common NAC protocol was a total dose of 2.4 g, administered as 600 mg twice a day on the day prior to and day of contrast administration. The oral route was most common (71 trials [70%]) followed by intravenous (30 [30%]) with 4 trials using both oral and intravenous. Additionally, in 1 trial NAC was administered intravenously and intrarenally. The most common control intervention was placebo, and the next most common was normal saline. Four trials^6,20,21,22,23^ compared NAC with 2 different control arms, and each of these was treated as separate data points for the quantitative synthesis.

### Primary Outcome: Contrast Induced Acute Kidney Injury

As shown in Table 2, NAC was associated with a significant benefit in the prevention of CI-AKI when all studies are considered together using either fixed-effects model or random-effects model, with the summary point estimate being more beneficial with a random effects model (pooled OR, 0.72; 95% CI, 0.63-0.82) than with a fixed effects model (pooled OR, 0.82; 95% CI, 0.76-0.90). However, there was significant statistical heterogeneity, with *I^2^* = 39 and *P* < .001, undermining the validity of these pooled estimates.

**Table 2.** NAC and Acute Kidney Injury Outcomes

Outcome	No. (%)	OR (95% CI)		Heterogeneity		Small study effect^a^
		Fixed effect	Random effect	Statistics, *I^2^*	*P* value	
CI-AKI, all studies	101 (100)	0.82 (0.76-0.90)	0.72 (0.63-0.82)	39	<.001	Yes
**Subgroup by sample size**						
CI-AKI, small studies (n < 200)	72 (71)	0.63 (0.54-0.74)	0.61 (0.51-0.74)	24	.04	Yes
CI-AKI, large studies (n ≥ 200)	29 (29)	0.92 (0.83-1.01)	0.85 (0.72-0.99)	49	<.001	Yes
CI-AKI, small studies (n < 500)	98 (97)	0.73 (0.66-0.82)	0.70 (0.66-0.81)	35.0	<.001	Yes
CI-AKI, large studies (n ≥ 500)	3 (3)	1.03 (0.89-1.18)	1.03 (0.89-1.18)	0	.93	No
**Subgroup by events**						
CI-AKI, small studies (n < 100)	95 (94)	0.69 (0.61-0.77)	0.67 (0.57-0.77)	33	<.001	Yes
CI-AKI, large studies (n ≥ 100)	6 (6)	1.02 (0.90-1.16)	1.02 (00.90-1.16)	0	.85	No
**Route of NAC**						
Oral	69 (68)	0.83 (0.75-0.92)	0.69 (0.58-0.82)	35	.003	Yes
Intravenous	32 (32)	0.81 (0.70-0.93)	0.76 (0.61-0.95)	48	<.001	Yes
**NAC dose**						
Low dose (<2.4 g total dose)	65 (64)	0.81 (0.72-0.90)	0.70 (0.59-0.82)	64	.02	Yes
High dose (>2.4 g total dose)	36 (36)	0.85 (0.75-0.96)	0.75 (0.60-0.93)	52	<.001	Yes

Abbreviations: CI-AKI, contrast induced acute kidney injury; NAC, N-acetylcysteine; OR, odds ratio.

^a^ Yes refers to evaluation based on funnel plot examination and the trim-and-fill analysis.

#### Subgroup Analyses Based on Sample Size and Events

The summary estimates of NAC showed a benefit, with statistical heterogeneity, when grouped based on a sample size of greater than 200 (fixed effects model: pooled OR, 0.92; 95% CI, 0.83-1.01; random effects model: pooled OR, 0.85; 95% CI, 0.72-0.99) or less than or equal to 200 (fixed effects model: pooled OR, 0.62; 95% CI, 0.54-0.74; random effects model: pooled OR, 0.61; 95% CI, 0.51-0.74), as well as in the subgroup with sample size less than or equal to 500 (fixed effects model: pooled OR, 0.73; 95% CI, 0.66-0.82; random effects model: pooled OR, 0.70; 95% CI, 0.66-0.81). However, when restricting to large studies that include more than 500 participants, NAC was not associated with a benefit in the prevention of CI-AKI and has a pooled OR of 1.03 (95% CI, 0.89-1.18) and resolution of heterogeneity (*I^2^* = 0). Similar findings were observed when stratifying trials by number of events (more than 100 compared with less than or equal to 100). In the subgroup of 95 trials with 100 events or less, NAC was associated with lower CI-AKI (fixed effects model: pooled OR, 0.69; 95% CI, 0.61-0.77; random effects model: pooled OR, 0.67; 95% CI, 0.57 to 0.77) with significant heterogeneity as well (*I^2^* = 33). However, in the subgroup of 6 trials with more than 100 events, there was no benefit with NAC (pooled OR, 1.03; 95% CI, 0.90-1.16) with lack of heterogeneity as well (*I^2^* = 0).

#### Subgroup Analyses With NAC, Publication Bias, and Small Study Effects

When grouped based on oral or intravenous NAC, or the dose of NAC (2.4g or less, and more than 2.4 g), all analyses showed a benefit associated with NAC on CI-AKI along with significant statistical heterogeneity (Table 2). The funnel plots of all trials that met inclusion criteria are shown in eFigure 1 and 2 in the Supplement. There was considerable asymmetry, suggesting the absence of small null trials of NAC for the prevention of CI-AKI, with a significant Egger statistic (*P* < .001). eFigure 2 in the Supplement shows that imputed analysis shifted the result toward the null. When the analysis was restricted to large studies with 500 or more participants, or to studies with 100 or more events of CI-AKI, there was no evidence of small-study effects on visual examination of the funnel plot, and the Egger statistic was not significant (*P* = .29 for studies with more than 500 participants and *P* = .97 for studies with more than 100 events).

### Clinical Outcomes: Kidney Replacement Therapy and Mortality

The odds of requiring KRT after CI-AKI was reported in 45 trials, and all-cause mortality was reported in 41 trials. Overall, the analyses demonstrated no benefit associated with NAC in preventing the need for either KRT following CI-AKI (pooled OR, 0.86; 95% CI, 0.69-1.12) or for preventing death (pooled OR, 0.93; 95% CI, 0.77-1.11). As seen Table 3, subgroup analysis by size of study yielded the same results, though the point estimate was toward the beneficial side in the subgroups with smaller sample size. There was no significant heterogeneity, publication bias, or small-study effects observed for most analyses for these outcomes.

**Table 3.** Kidney Replacement Therapy and Mortality Outcomes

Outcome	No.	OR (95% CI)		Heterogeneity		Publication bias and small-study effect?^a^
		Fixed effect	Random effect	Statistics, I^2^	*P* value	
**Need for KRT**						
All studies	45	0.86 (0.66-1.12)	0.86 (0.66-1.12)	0	>.99	No
Small studies (n ≤ 200)	30	0.71 (0.46-1.09)	0.71 (0.46-1.09)	0	.98	No
Large studies (n > 200)	15	0.97 (0.69-1.35)	0.97 (0.69-1.35)	0	>.99	No
**Mortality**						
All studies	41	0.93 (0.77-1.11)	0.93 (0.77-1.11)	0	.98	Yes
Small studies (n ≤ 200)	23	0.89 (0.61-1.30)	0.89 (0.61-1.30)	0	>.99	No
Large studies (n > 200)	18	0.94 (0.76-1.16)	0.94 (0.76-1.16)	0	>.99	No

Abbreviations: KRT, kidney replacement therapy; OR, odds ratio.

^a^ Yes refers to evaluation based on funnel plot examination and the trim-and-fill analysis.

### Metaregression

The univariate metaregression demonstrates that the total sample size and number of events were significantly associated with outcome effect size, with less benefit with NAC being seen in larger studies based on either sample size or total number of events (eTable 2 in the Supplement). The metaregression was not significant for the other covariates studied, including route of contrast, route of NAC, dose of NAC, baseline creatinine, year of publication, and select study level demographic variables.

### Risk of Bias Assessment

eFigure 3 in the Supplement shows the risk of bias assessment for all included trials. More than 50% of trials were judged to be at least at unclear risk of bias with respect to random sequence generation, allocation concealment, blinding of participants and personnel, and blinding of outcome assessment. More than 25% of trials were deemed to be at high risk of reporting bias because of selective reporting of outcomes.

## Discussion

We conducted a systematic review and meta-analysis on role of NAC in preventing CI-AKI and its clinical sequelae, mainly the need to KRT and all-cause mortality. Despite the large number of trials in this area the pooled effect shows a misleading beneficial association of NAC with the prevention of CI-AKI. This is the case both when fixed and random effects models were used. However, there is significant heterogeneity between studies and evidence of publication bias, which together undermine the reliability of the pooled effect measured for all trials. These issues were minimized only when we restricted the analysis to large studies, with a sample size of 500 or more. Indeed, the large trials were homogeneous (*I^2^* = 0%) and did not show a benefit for NAC in preventing CI-AKI. Similar findings were observed when we stratified trials by number of CI-AKI events, to trials with more than 100 events. Finally, restricting the analysis to trials with clinical outcomes, such as the need for KRT after CI-AKI or all-cause mortality, also provided a more robust, and neutral, summary result with no heterogeneity or publication bias.

NAC and CI-AKI in this meta-analysis exemplifies how more than 100 trials can be performed over 2 decades before a reversal in practice occurs. NAC is an inexpensive and easily available and administered drug, so it is easy to understand its widespread use after the publication of a single influential trial^4^ of 80 patients with a surrogate CI-AKI outcome. However, many subsequent small trials were then conducted to replicate these findings with mixed results. As we demonstrate in this meta-analysis, the 2 decades of confusion and incorrect practice could have been shortened if trials with a large sample size, a sufficient number of CI-AKI events, or one powered for clinically relevant outcomes had been conducted. While a handful of small trials with surrogate outcomes are useful for hypothesis generation and proof of concept exploration, we found 69 trials with less than 200 participants published on this question since 2000. These small trials cloud the picture when large trials with clinical outcomes were needed to demonstrate a clinically significant result.

Small study effect has been reported earlier in the form of a larger association being reported in studies with smaller sample size.^24,25,26^ For example, a 2013 meta-epidemiological study^25^ found that, on average, treatment effects were 48% larger in trials with fewer than 50 patients compared with trials with more than 1000 patients (ratio of odds ratios, 0.52; IQR, 0.41-0.66). However, these studies do not inform us of the true magnitude of association with the reported interventions. In the case of NAC and CI-AKI, we know that this is a null effect from the well-designed large trials that reported clinical outcomes.^3,27^ This allowed us to explore which analytic methods provide summary effects closer to the truth, which is also supported statistically by resolution of statistical heterogeneity and absence of publication bias.

Apart from study size, we also demonstrated that biochemical CI-AKI is not a reliable surrogate end point for clinical trials. In the AKI research area, definitions of AKI have varied a lot over the years,^28^ and only recently have become more standardized. Additionally, in a substudy of the Prevention of Serious Adverse Events following Angiography, or PRESERVE, trial, 922 participants underwent measurement of biomarkers of tubular injury and repair.^29^ There were no significant differences in either the absolute change or relative ratios of injury and repair biomarkers with AKI after contrast administration, possibly as small changes in creatinine as picked up by these AKI definitions represent changes in volume status and hemodynamics rather than true kidney injury. Lastly, in a systematic review^30^ of 14 trials that reported a benefit with an intervention on creatinine-based AKI outcome, no change was seen in longer term clinical outcomes, such as development of chronic kidney disease or mortality. Hence the biochemical AKI outcome may be reasonable to use for an initial pilot or feasibility trial, but practice should not change until definitive trials with meaningful clinical outcomes are conducted. For AKI, major adverse kidney events at 90 days following AKI (ie, MAKE90), a new-onset need for KRT, and death would be a suitable end point.^31^

The discrepancy in benefit with NAC observed between studies that use CI-AKI defined as a change in creatinine levels compared with those that use clinical outcomes has also been explored elsewhere. In a systematic review, Huang et al^32^ showed that NAC causes a decrease in serum creatinine but not in cystatin C suggesting analytic interference as opposed to any true effect on kidney function. This effect was greater when the enzymatic assay was used to estimate creatinine compared with the Jaffe colorimetric method. These findings are consistent with a subsequent in vitro analysis showing that NAC interferes with, by lowering, plasma creatinine as measured only by the enzymatic assay and not the Jaffe method.^33^ This effect appears to be dose-dependent and is greater than 10% at NAC concentrations greater than 400 ug/L. Huang et al^32^ similarly showed that intravenous NAC administration has a greater effect on serum creatinine than oral administration, which might be because of higher achieved NAC concentrations and greater interference. Though the decrease in serum creatinine with oral NAC may not be clinically significant, it may be adequate to show difference in CI-AKI when defined by a change in serum creatinine. Therefore, it is not surprising that a change in serum creatinine is a surrogate marker that only imprecisely estimates direct kidney damage. This aspect adds an additional layer of complexity, which may have resulted in the confusing and heterogeneous literature around NAC and CI-AKI.

One might question the use of our findings when the large, definitive randomized control trials on NAC for the prevention of CI-AKI have already demonstrated that it has no benefit.^3^ What we were able to show, in addition to the aspects discussed above, is that standard meta-analytic techniques to evaluate a body of literature replete with small trials that assess surrogate rather than clinical outcomes can be dangerous and misleading. In the face of significant statistical heterogeneity, meta-analyses often present random-effects analysis as a solution, which we demonstrated here to be far from a robust solution. Indeed, when all the literature on this question was pooled, a standard meta-analysis resulted in a beneficial result, which we know from the large trials to be incorrect. This is important because the regulatory shift has been toward approving drugs based on surrogate biomarkers. In the chronic kidney disease field, albuminuria and the slope of kidney function have become acceptable as valid surrogates.^34^ The definitions of AKI were primarily developed to aid in clinical research and provide valid surrogate outcomes.^35,36^ Surrogate markers play an important role in generating hypotheses and answering questions when clinical outcomes are rare; however, as we have shown, they are not without limitation. What we demonstrate is that these may not be sufficient, and if drugs are approved based on such surrogate outcomes, follow-up studies should be mandated with relevant clinical outcomes.

### Limitations

This study had limitations. Despite being a systematic review of RCTs, this is an observational study, and the inferences on the association between sample size and outcome choice are correlative rather than definitive. The metaregression we report are at a study level and not individual patient level and susceptible to ecological fallacy. Lastly, the findings of using a certain threshold for study size may not be generalizable across other studies in the medical field. Nevertheless, the clear difference between the surrogate outcomes (ie, CI-AKI by change in creatinine) and the clinical outcomes (ie, the need for KRT) is likely to be valid in the nephrology literature.

## Conclusions

In this study, NAC was not associated with any benefit in the prevention of CI-AKI, which was known from large trials already. However, standard meta-analytic techniques report a spurious benefit of NAC for AKI when applied to all of the literature because of the trial size, choice of outcomes, quality issues, and publication bias. More robust and neutral effect sizes are found when publication bias is accounted for and analysis is restricted to RCTs with a large sample size or trials with clinical outcomes over surrogate biomarkers reduces this issue.

## Notes

Supplement. **eAppendix.** Database Search Strategies **eTable 1.** All Studies Used in Systematic Review **eTable 2.** Univariate Metaregression **eFigure 1.** Funnel Plot of All Trials **eFigure 2.** Funnel Plot of Observed and Imputed Trials **eFigure 3.** Risk of Bias Assessment
