# Supplementary material for: Meta-analytic Techniques to Assess the Association Between N-acetylcysteine and Acute Kidney Injury After Contrast Administration: A Systematic Review and Meta-analysis
Source: JAMA Netw Open. 2022 Jul 5;5(7):e2220671. doi: 10.1001/jamanetworkopen.2022.20671 (PMC9257561; doi:10.1001/jamanetworkopen.2022.20671)
Supplement: Supplement. — eAppendix. Database Search Strategies eTable 1. All Studies Used in Systematic Review eTable 2. Univariate Metaregression eFigure 1. Funnel Plot of All Trials eFigure 2. Funnel Plot of Observed and Imputed Trials eFigure 3. Risk of Bias Assessment [file jamanetwopen-e2220671-s001.pdf]

Supplemental Online Content

Magner K, Ilin JV, Clark EG, Kong JWY, Davis A, Hiremath S. Meta-analytic techniques to assess the association between N-acetylcysteine and acute kidney injury after contrast administration: a systematic review and meta-analysis. *JAMA Netw Open*. 2022;5(7):e2220671. doi:10.1001/jamanetworkopen.2022.20671

eAppendix. Database Search Strategies

eTable 1. All Studies Used in Systematic Review

eTable 2. Univariate Metaregression

eFigure 1. Funnel Plot of All Trials

eFigure 2. Funnel Plot of Observed and Imputed Trials

eFigure 3. Risk of Bias Assessment

This supplemental material has been provided by the authors to give readers additional information about their work.

© 2022 Magner K et al. *JAMA Network Open.*

eAppendix. Database Search Strategy

Database: Ovid MEDLINE(R) ALL

Search Strategy:

----

1 Acetylcysteine/
2 (acetylcysteine or "n-acetyl-l-cysteine" or "n-acetyl cysteine" or NAC or acetadote or fluimucil or mucomyst).tw,kf.
3 1 or 2
4 exp Contrast Media/
5 (contrast media or contrast medium or contrast material$ or contrast agent$ or contrast procedure* or contrast dye or contrast exposure or contrast-based or contrast induced or contrast imag* or radiographic contrast).tw,kw.
6 (radiocontrast or radio contrast).tw,kw.
7 (ioxithalamate or meglumine or iopamidol or iohexol or iopromide or iotrolan or iodixanol or ioversol).tw,kw.
8 exp Triiodobenzoic Acids/
9 Cardiac Catheterization/ or cardiac catheterization.tw,kw.
10 exp Tomography, X-Ray Computed/ or (computed tomograph* or ct scan*).tw,kw.
11 exp Angiography/ or angiography.tw,kw.
12 exp Percutaneous Coronary Intervention/ or (percutaneous coronary intervention or PCI).tw,kw.
13 Acute Kidney Injury/ci [Chemically Induced]
14 (contrast-induced nephr$ or contrast-associated nephr$).tw,kw.
15 (Contrast-induced acute kidney injur$ or ci-aki or ciaki or caaki).tw,kw.
16 cin.tw,kw.
17 or/4-16
18 randomized controlled trial.pt.
19 clinical trial.pt.
20 randomi?ed.ti,ab.
21 placebo.ti,ab.
22 randomly.ti,ab.
23 trial.ti.
24 18 or 19 or 20 or 21 or 22 or 23
25 3 and 17 and 24
26 (child/ or infant/) not adult/
27 25 not 26

28 meta analysis.pt.
29 (meta analys* or metaanalys*).tw.
30 28 or 29
31 3 and 17 and 30

Database: Embase Classic+Embase
Search Strategy:

----

1 *acetylcysteine/
2 (acetylcysteine or "n-acetyl-l-cysteine" or "n-acetyl cysteine" or NAC or acetadote or fluimucil or mucomyst).tw.
3 1 or 2

© 2022 Magner K et al. *JAMA Network Open.*

4 exp contrast medium/
5 (contrast media or contrast medium or contrast material$ or contrast agent$ or contrast procedure* or contrast dye or contrast exposure or contrast-based or contrast induced or contrast imag* or radiographic contrast).tw.
6 (radiocontrast or radio contrast).tw.
7 (ioxithalamate or meglumine or iopamidol or iohexol or iopromide or iotrolan or iodixanol or ioversol).tw.
8 heart catheterization/
9 cardiac catheterization.tw.
10 angiography/
11 angiography.tw.
12 computer assisted tomography/
13 (computed tomograph* or ct scan*).tw.
14 percutaneous coronary intervention/
15 (percutaneous coronary intervention or PCI).tw.
16 contrast induced nephropathy/ or (contrast-induced nephr$ or contrast-associated nephr$).tw.
17 ((impair$ or damag$ or reduc$ or dysfunction or injur$ or insufficienc$) adj2 (renal or kidney)).tw.
18 (Contrast-induced acute kidney injur$ or ci-aki or ciaki or caaki).tw.
19 or/4-18
20 3 and 19
21 double-blind procedure/ or randomized controlled trial/ or single-blind procedure/ or (random* or factorial* or crossover* or cross over* or placebo* or (doubl* adj blind*) or (singl* adj blind*) or assign* or allocat* or volunteer*).tw.
22 20 and 21
23 (child/ or infant/) not adult/
24 22 not 23
25 (animal/ or animal experiment/ or animal model/) not human/
26 24 not 25

27 meta analysis/
28 (meta analys* or metaanalys*).tw.
29 27 or 28
30 24 and 29

Database: EBM Reviews - Cochrane Central Register of Controlled Trials
Search Strategy:
----
1 Acetylcysteine/
2 (acetylcysteine or "n-acetyl-l-cysteine" or "n-acetyl cysteine" or NAC or acetadote or fluimucil or mucomyst).tw.
3 1 or 2
4 exp Contrast Media/
5 (contrast media or contrast medium or contrast material$ or contrast agent$ or contrast procedure* or contrast dye or contrast exposure or contrast-based or contrast induced or contrast imag* or radiographic contrast).tw.
6 (radiocontrast or radio contrast).tw.
7 (ioxithalamate or meglumine or iopamidol or iohexol or iopromide or iotrolan or iodixanol or ioversol).tw.
8 exp Triiodobenzoic Acids/
9 Cardiac Catheterization/ or cardiac catheterization.tw.
10 exp Tomography, X-Ray Computed/ or (computed tomograph* or ct scan*).tw.

© 2022 Magner K et al. *JAMA Network Open.*

11 exp Angiography/ or angiography.tw.
12 exp Percutaneous Coronary Intervention/ or (percutaneous coronary intervention or PCI).tw.
13 (contrast-induced nephr$ or contrast-associated nephr$).tw.
14 (Contrast-induced acute kidney injur$ or ci-aki or ciaki or caaki).tw.
15 cin.tw.
16 or/4-15
17 3 and 16

© 2022 Magner K et al. *JAMA Network Open.*

eTable 1. All Studies Used in Systematic Review

	Authors	Year	Country	Total Sample size	Treatment studied	Control	Comparator arm sample size	Number of events (%)	Mean age	Sex (%M)	Intervention arm sample size	Number of events (%)	Mean age	Sex (%M)
1	Agrawal et al.	2004	USA	25	Coronary Angiography	NS + placebo	14	14	62.8	57	11	18	65.5	82
2	Albabtain et al.	2013	Saudi Arabia	128	Coronary Angiography	NS + placebo	66	6.2	59.8	81.8	62	6.8	62	71
3	Alessandri et al.	2013	Italy	296	Coronary Angiography	Placebo	158	9.4	65.1	67.7	138	7.2	64.3	66.7
4	Alioglu et al.	2013	Turkey	113	Coronary Angiography +/- PCI	NS + placebo	64	17.2	60.8	65.6	49	12.2	62.7	67.3
5	Allaqaband et al.	2002	USA	85	Coronary Angiography	Placebo	40	15.3	71	60	45	17.7	70	62.2
6	Amini et al.	2009	Iran	90	Coronary Angiography	NS + placebo	45	14.3	65.1	75.6	45	11.1	63.3	80
7	Arabmomeni et al.	2014	Iran	58	Coronary Angiography +/- PCI	Theophylline	30	20	65	43.3	28	7.1	64.5	39.3
8	Aslanger et al.	2012	Turkey	411	Coronary Angiography +/- PCI	Placebo	198	23.2	57.2	74	213	24	56.1	79
9	Baker et al.	2003	United Kingdom	80	Coronary Angiography +/- PCI	Placebo	39	20.5	70.9	84.6	41	4.9	67.4	90.2
10	Balderramo et al.	2004	Argentina	61	Coronary Angiography +/- PCI	NS + placebo	28	7.1	69.7	64.3	33	3	71.1	69.7
11	Baranska-Kosakowska et al.	2007	Poland	112	Coronary Angiography	NS + placebo	57	N/A	52	89	55	0	55	91
12	Baskurt et al.	2009	Turkey	145	Coronary Angiography	Placebo	72	6.9	67.1	56.9	73	9.6	67.9	63

© 2022 Magner K et al. *JAMA Network Open.*

	Authors	Year	Country	Total Sample size	Treatment studied	Control	Comparator arm sample size	Number of events (%)	Mean age	Sex (%M)	Intervention arm sample size	Number of events (%)	Mean age	Sex (%M)
13	Berwanger et al.	2013	Brazil	1394	Coronary Angiography	Placebo	678	14.7	64.3	59.3	716	13.8	64.6	60.8
14	Biernacka - Fialkowska et al.	2018	Poland	222	Coronary Angiography +/- PCI	NS + placebo	114	18.4	64.3	72.8	108	8.3	66	81.5
15	Boccalandro et al.	2003	USA	179	Coronary Angiography	NS + placebo	106	12	65	55.6	73	13	66	67.1
16	Brajkovic et al.	2019	Croatia	69	Coronary Angiography	NaHCO3	40	0	67.5	70	29	0	71	76
17	Briguori et al.	2002	Italy	183	Coronary + peripheral angiography	Placebo	91	11	64	89	92	6.5	64	84
18	Briguori et al.	2004	Italy	192	Coronary Angiography	Fenoldopam	95	13.7	69	83	97	4.1	68	87
19	Brueck et al.	2013	Germany	385	Coronary Angiography +/- PCI	Placebo	193	32.1	74	62.1	192	27.6	75	65.3
20	Burns et al.	2010	Canada	42	CT	D5W	21	14.3	N/A	N/A	21	4.8	N/A	N/A
21	Buyukhatipoglu et al.	2010	Turkey	60	Coronary Angiography +/- PCI	NS + placebo	30	N/A	61.8	70	30	3.3	58.9	70
22	Caglar et al.	2014	Russia	100	Coronary Angiography +/- PCI	NaHCO3	50	0	68.3	32	50	0	67.2	72
23	Carbonell et al.	2007	Spain	216	Coronary Angiography	Placebo	109	10.1	60.7	72.5	107	10.3	63.1	80.4

© 2022 Magner K et al. *JAMA Network Open.*

	Authors	Year	Country	Total Sample size	Treatment studied	Control	Comparator arm sample size	Number of events (%)	Mean age	Sex (%M)	Intervention arm sample size	Number of events (%)	Mean age	Sex (%M)
24	Carbonell et al.	2010	Spain	81	Coronary Angiography	NS + placebo	42	23.8	53	81	39	5.1	70	80
25	Castini et al.	2010	Italy	104	Coronary Angiography +/- PCI	Placebo	51	14	72.7	84	53	17	70.5	94
26	Chong et al.	2015	Singapore	309	Coronary Angiography +/- PCI	NaHCO3	153	12.8	68.4	77.8	156	10.6	67	77.6
27	Chouster man et al.	2013	France	140	CT/angio	NS + placebo	70	21	N/A	N/A	70	14	N/A	N/A
28	Coyle et al.	2006	USA	137	Coronary Angiography	Placebo	69	1.4	63.3	68.1	68	9.2	66.7	61.8
29	Demir et al.	2008	Turkey	40	CT	Placebo	20	0	58.2	75	20	5	62	55
30	Diaz-Sandoval et al.	2002	USA	54	Coronary Angiography	NS + placebo	29	45	72	90	25	8	74	72
31	Droppa et al.	2011	Germany	251	Coronary Angiography +/- PCI	Placebo	125	70.4	65	66	126	74.6	66	71
32	Durham et al.	2002	USA	79	Coronary Angiography	NS + placebo	41	22	69.8	68.2	38	26.3	71.4	63.2
33	Efrati et al.	2003	USA	49	Coronary Angiography	NS + coca cola	25	3.4	66	92	24	0	68	87.5
34	Erturk et al.	2014	Turkey	205	Coronary + peripheral angiography	Placebo	103	6.8	67	63.1	102	13.7	65	62.7

© 2022 Magner K et al. *JAMA Network Open.*

	Authors	Year	Country	Total Sample size	Treatment studied	Control	Comparator arm sample size	Number of events (%)	Mean age	Sex (%M)	Intervention arm sample size	Number of events (%)	Mean age	Sex (%M)
35	Ferrario et al.	2009	Italy	200	Coronary Angiography +/- PCI	NS + placebo	101	5.9	75	62	99	8.1	75	68
36	Fung et al.	2004	Hong Kong	91	Coronary Angiography +/- PCI	Placebo	45	13.3	68	66.7	46	17.4	68.2	73.9
37	Garcia et al.	2018	USA	1161	Coronary Angiography +/- PCI	Placebo	563	11.7	69.1	N/A	598	11.5	69.5	N/A
38	Goldenberg et al.	2004	Israel	80	Coronary Angiography +/- PCI	NS + placebo	39	8	69	79.5	41	10	71	85.4
39	Gomes et al.	2005	Brazil	156	Coronary Angiography +/- PCI	NS + placebo	79	10.1	84.8	57	77	10.4	63.8	61
40	Gulel et al.	2005	Turkey	50	Coronary Angiography	Placebo	25	8	69	72	25	12	61.4	80
41	Gunebakmaz et al.	2012	Turkey	80	Coronary Angiography	Placebo	40	27.5	66.4	62.5	40	22.5	66.4	72.5
42	Habib et al.	2016	Palestine	75	Coronary Angiography	Placebo	45	17.8	63	N/A	30	6.66	63	66.6
43	Hafiz et al.	2012	USA	161	Coronary Angiography	Placebo	80	13.8	73	43.1	81	9.9	73	43.1
44	Heguilen et al.	2013	Argentina	85	Coronary + peripheral angiography	$NaHCO_3$	42	35.7	67.7	64.3	43	7	64.8	74.4
45	Holscher et al.	2008	Germany	278	Coronary Angiography	Placebo	139	7.2	67	83.5	139	4.3	67	83.5

© 2022 Magner K et al. *JAMA Network Open.*

	Authors	Year	Country	Total Sample size	Treatment studied	Control	Comparator arm sample size	Number of events (%)	Mean age	Sex (%M)	Intervention arm sample size	Number of events (%)	Mean age	Sex (%M)
46	Hsu et al.	2012	Taiwan	209	CT	Placebo	103	19.4	79.3	75.7	106	11.3	79.7	73.6
47	Huber et al.	2006	Hungary	100	CT	Theophylline	51	2	59.6	N/A	49	4	60.6	N/A
48	Inda-Filho et al.	2014	Brazil	251	Coronary Angiography	Placebo	125	11.2	60.5	58.4	126	7.1	59.2	61.9
49	Jaffery et al.	2012	USA	398	Coronary Angiography	NS + placebo	192	13	65.1	59.4	206	16	65.6	67
50	Jo et al.	2009	South Korea	212	Coronary Angiography +/- PCI	Ascorbic acid	106	4.4	65.6	73.6	106	1.2	64.3	82.1
51	Kama et al.	2014	Turkey	71	CT	Placebo	35	14.2	67	67.3	36	19.4	69	59.4
52	Kay et al.	2003	Hong Kong	200	Coronary Angiography	NS + placebo	98	12	69	63	102	4	69	60
53	Kefer et al.	2003	Belgium	104	Coronary Angiography	NS + placebo	51	5.9	64	76	53	3.8	61	77
54	Khalili et al.	2006	Iran	70	CT	Placebo	35	34.3	55.9	62.9	35	14.3	59.8	57.1
55	Kim et al.	2010	South Korea	166	Coronary Angiography	Placebo	86	8.1	62	58	80	3.8	62	63
56	Kimmel et al.	2008	Germany	36	Coronary Angiography	Placebo	17	5.5	66.8	70	19	N/A	71.5	79
57	Kinbara et al.	2010	Japan	30	Coronary Angiography +/- PCI	Placebo	15	26.7	70	60	15	0	71	66.7

© 2022 Magner K et al. *JAMA Network Open.*

	Authors	Year	Country	Total Sample size	Treatment studied	Control	Comparator arm sample size	Number of events (%)	Mean age	Sex (%M)	Intervention arm sample size	Number of events (%)	Mean age	Sex (%M)
58	Kitzler et al.	2012	Canada	20	Coronary Angiography	NS + placebo	10	0	74	50	10	0	76.6	20
59	Koc et al.	2012	Turkey	140	Coronary Angiography	Placebo	60	16.3	65	79	80	2.5	62	76
60	Kotlyar et al.	2005	Australia	60	Coronary + peripheral angiography	Placebo	19	11	69	89	41	15	66	80.5
61	Kumar et al.	2014	India	180	Coronary Angiography +/- PCI	Placebo	90	34.4	78	65	90	20	78	65
62	Lawlor et al.	2007	Canada	50	Coronary Angiography	NS + placebo	25	8	N/A	68	25	8	N/A	76
63	MacNeill et al.	2003	USA	43	Coronary Angiography	NS + placebo	22	32.2	72.9	95.4	21	4.8	72.5	76.2
64	Marenzi et al.	2006	Italy	354	Peripheral angiography + angioplasty	NS + placebo	238	32.8	62.6	82	116	11.6	62.5	80.5
65	Miner et al.	2004	Canada	180	Coronary Angiography +/- PCI	NS + placebo	85	22.2	69	66	95	9.6	71	68
66	Momeni et al.	2012	Iran	110	IVP/CR	NS + placebo	55	0	42.7	47.3	55	N/A	45.3	47.3
67	Moore et al.	2006	United Kingdom	20	EVAR	NS + placebo	9	0	72	100	11	0	72	100
68	Ng et al.	2006	USA	95	Coronary Angiography	Fenoldopam + NS	47	20	69	70.2	48	11.4	67	81.2

© 2022 Magner K et al. *JAMA Network Open.*

	Authors	Year	Country	Total Sample size	Treatment studied	Control	Comparator arm sample size	Number of events (%)	Mean age	Sex (%M)	Intervention arm sample size	Number of events (%)	Mean age	Sex (%M)
69	Ochoa et al.	2004	USA	80	Coronary Angiography	NS + placebo	44	25	70	41	36	8	73	44
70	Oldemeyer et al.	2003	USA	96	Coronary Angiography	NS + placebo	47	6.4	75	53.1	49	8.2	77	55.1
71	Ozcan et al.	2007	Turkey	176	Coronary Angiography +/- PCI	Placebo	88	13.6	70	75	88	12.5	67	76.1
72	Palli et al.	2017	Greece	124	CT	Placebo	64	15.6	50.5	85.9	60	18.3	51.3	76.7
73	Park et al.	2016	South Korea	168	Coronary Angiography +/- PCI	NS + Statin	84	17.9	60.6	77.4	84	14.3	64.5	77.4
74	Pezeshgi et al.	2015	Iran	150	Coronary Angiography	Placebo	75	17.3	N/A	N/A	75	1.33	N/A	N/A
75	Poletti et al.	2007	Switzerland	100	CT	Placebo	50	21	72.7	67	50	5	69.5	59
76	Poletti et al.	2013	Switzerland	114	CT	NS + placebo	59	22	78.2	49.2	55	27	78.1	50.9
77	Rashid et al.	2004	United Kingdom	94	Peripheral angiography + angioplasty	NS + placebo	48	14.3	68.8	68.8	46	17.6	72	58.7
78	Ratcliffe et al.	2009	USA	36	Coronary Angiography +/- PCI	Placebo	15	7	64	60	21	5	65	52
79	Recio-Mayoral et al.	2007	United Kingdom	111	Coronary Angiography +/- PCI	Placebo	55	21.8	64	71	56	1.8	65	68

© 2022 Magner K et al. *JAMA Network Open.*

	Authors	Year	Country	Total Sample size	Treatment studied	Control	Comparator arm sample size	Number of events (%)	Mean age	Sex (%M)	Intervention arm sample size	Number of events (%)	Mean age	Sex (%M)
80	Reinecke et al.	2007	Germany	286	Coronary Angiography	Placebo	140	6.1	66.7	82.9	146	5.3	66.7	82.9
81	Sadat et al.	2011	United Kingdom	40	Peripheral angiography + angioplasty	Placebo	19	15.8	70.5	N/A	21	4.8	74.5	N/A
82	Sadineni et al.	2017	India	65	Coronary Angiography +/- PCI	NS + placebo	30	36	62.6	86.7	35	20	60.7	77.1
83	Saitoh et al.	2011	Japan	14	Coronary Angiography	Placebo	7	14.1	76.5	86	7	14.1	72.1	86
84	Samadi et al.	2020	Iran	201	Coronary Angiography	NS + placebo	63	0	N/A	45.8	138	0	N/A	29.7
85	Sandhu et al.	2006	United Kingdom	106	Coronary Angiography	NS + placebo	53	0	66	59	53	5.6	69.3	66
86	Sar et al.	2010	Italy	45	CT	Placebo	20	15	53.5	55	25	0	60	52
87	Seyon et al.	2007	Canada	40	Coronary Angiography +/- PCI	NS + placebo	20	5	74.7	70	20	2.5	76.4	60
88	Shavit et al.	2009	Israel	87	Coronary Angiography +/- PCI	NaHCO3	51	9.8	72	84	36	8.4	71	70
89	Shyu et al.	2002	Taiwan	121	Coronary Angiography	NS + placebo	61	24.6	70	65.6	60	3.3	70	70
90	Tanaka et al.	2011	Japan	76	Coronary Angiography +/- PCI	Placebo	38	13.2	60.5	82	38	5.3	62.8	82
91	Tepel et al.	2000	Germany	83	CT	NS + placebo	42	21	65	54.8	41	2	66	58.5

© 2022 Magner K et al. *JAMA Network Open.*

	Authors	Year	Country	Total Sample size	Treatment studied	Control	Comparator arm sample size	Number of events (%)	Mean age	Sex (%M)	Intervention arm sample size	Number of events (%)	Mean age	Sex (%M)
92	Thayssen et al.	2014	Denmark	357	Coronary Angiography +/- PCI	Placebo	181	26.5	63	80.1	176	20.1	63	72.2
93	Thiele et al.	2010	Germany	251	Coronary Angiography +/- PCI	NS + placebo	125	20	68	66	126	14	68	71
94	Traub et al.	2013	USA	399	CT	Placebo	199	7	59.7	43	200	7.6	61.5	38
95	Turedi et al.	2016	Turkey	172	CTPA	Placebo	87	26.4	74	52.9	85	23.5	76	48.2
96	Usmiani et al.	2016	Italy	124	Coronary Angiography +/- PCI	Placebo	65	25	75	71	59	7	76	78
97	Wang et al.	2008	Taiwan	46	Coronary Angiography	NS + placebo	23	0	69.26	60.9	23	N/A	65.9	56.5
98	Webb et al.	2004	Canada	487	Coronary Angiography +/- PCI	NS + placebo	245	20.7	70.4	62	242	23.3	70.4	59.5
99	Weisbord et al.	2018	USA	4993	Coronary + peripheral angiography	NS + placebo + $NaHCO_3$	2498	8.7	69.6	93	2495	9.1	70	94
100	Yang et al.	2014	China	627	Coronary Angiography +/- PCI	Placebo	161	3.11	59.7	53.5	157	4.9	58.7	54.7
101	Yeganehkhah et al.	2014	Iran	100	Coronary Angiography	Placebo	50	14	N/A	44	50	12	60	50

© 2022 Magner K et al. *JAMA Network Open.*

eTable 2. Univariate Metaregression

Variable	Beta coefficient (95% CI)	p value
Change in creatinine	-0.75 (0.56 to -1.84)	0.17
**Total sample N**	**0.0016 (0.0005 to 0.0027)**	**0.006**
**Total event N**	**0.0014 (0.0001 to 0.0028)**	**0.034**
Contrast route	0.11 (-0.81 to 1.04)	0.81
NAC route	0.17 (-0.14 to 0.47)	0.28
NAC dose	0.03 (-0.02 to 0.10)	0.20
Baseline creatinine	-0.35 (-0.74 to 0.04)	0.08
Mean age	-0.0032 (-0.032 to 0.0257)	0.83
Men (%)	-0.0125 (-0.0253 to 0.0003)	0.055
DM (%)	-0.0013 (-0.0108 to 0.0081)	0.79
Year of publication	0.03 (-0.035 to 0.064)	0.08

The beta coefficient in this meta-regression describes the change in the odds ratio with a unit increase in the explanatory variable at a study level.

© 2022 Magner K et al. *JAMA Network Open.*

eFigure 1. Funnel Plot of All Trials

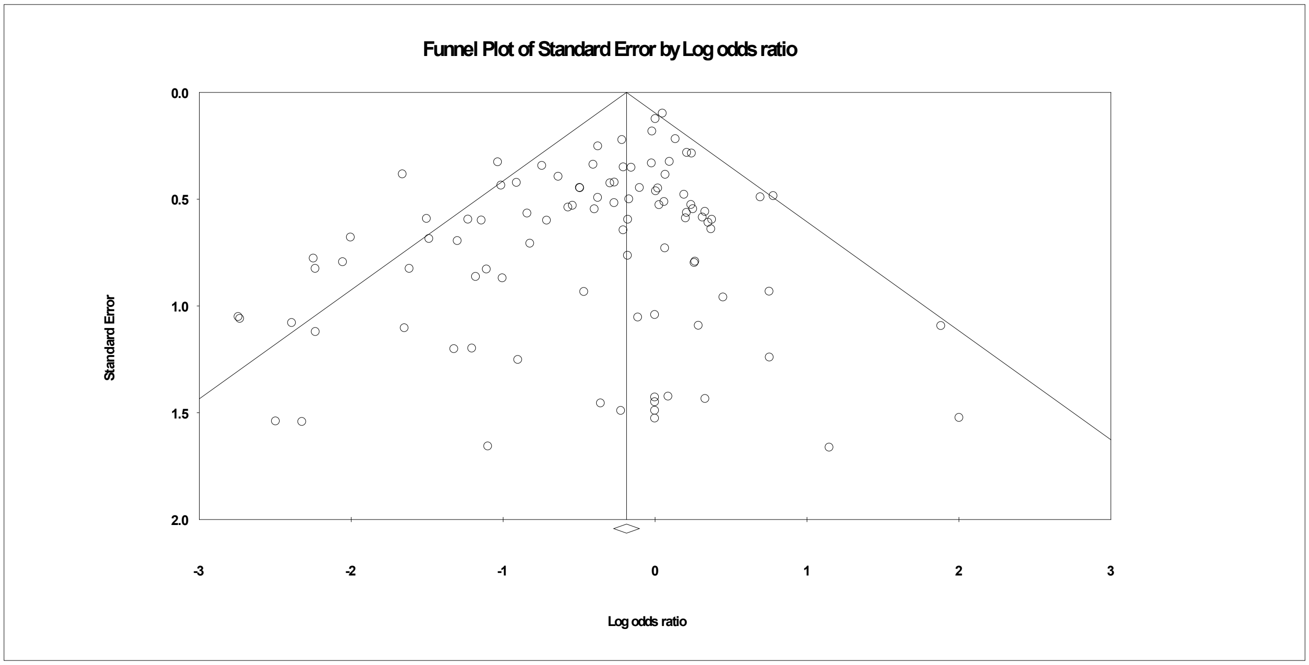

This is a funnel plot which includes all trials which reported CI-AKI outcomes. On visual examination, there is a paucity of studies in the lower right corner, suggesting a small study effect or possible publication bias.

© 2022 Magner K et al. *JAMA Network Open.*

eFigure 2. Funnel Plot of Observed and Imputed Trials

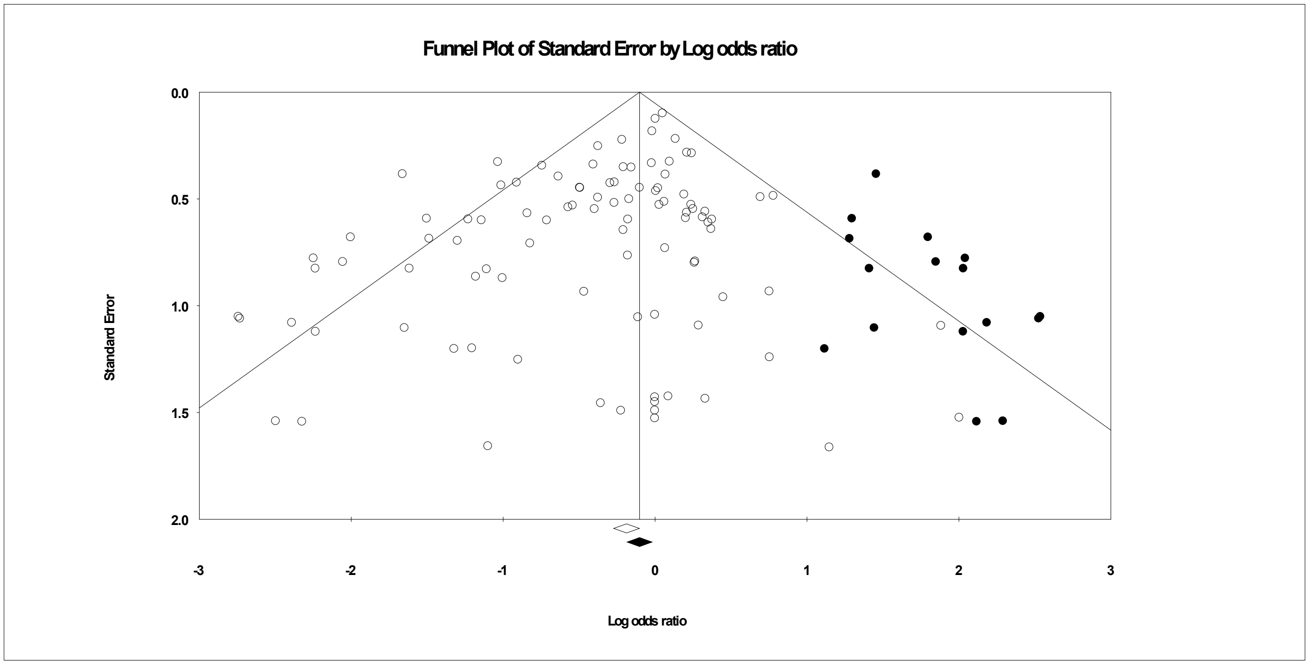

This is a funnel plot of observed (open circles) and imputed studies (black circles) for all trials, with CI-AKI as outcome. The imputed studies (black circles) demonstrates that this method imputed additional studies to force symmetry into the visual funnel plot.

© 2022 Magner K et al. *JAMA Network Open.*

eFigure 3. Risk of Bias Assessment

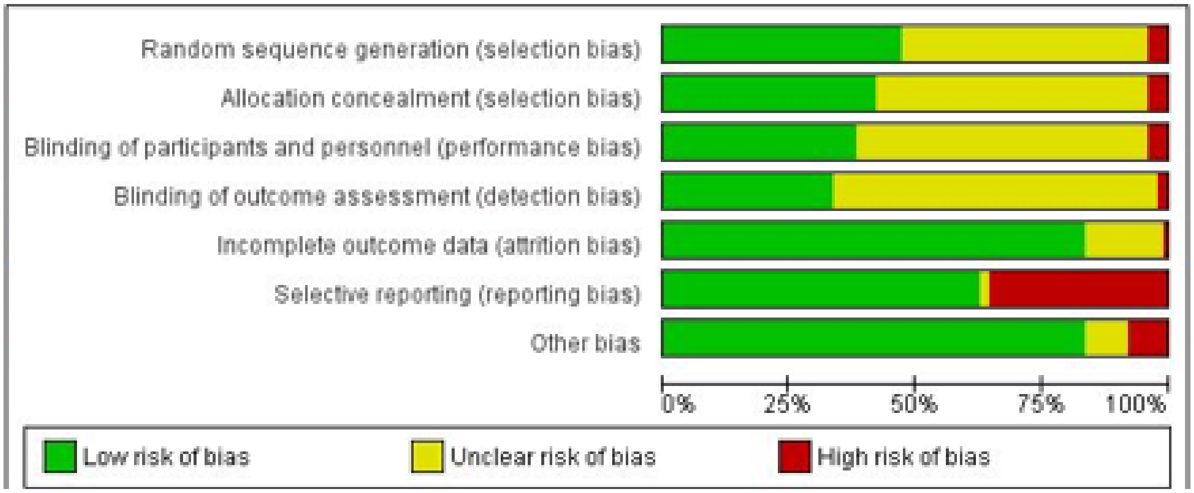

© 2022 Magner K et al. *JAMA Network Open.*
